# Projected Health Outcomes Associated With 3 US Supreme Court Decisions in 2022 on COVID-19 Workplace Protections, Handgun-Carry Restrictions, and Abortion Rights

**DOI:** 10.1001/jamanetworkopen.2023.15578

**Published:** 2023-06-08

**Authors:** Adam Gaffney, David U. Himmelstein, Samuel Dickman, Caitlin Myers, David Hemenway, Danny McCormick, Steffie Woolhandler

**Affiliations:** 1 Harvard Medical School, Boston, Massachusetts; 2 Department of Medicine, Cambridge Health Alliance, Cambridge, Massachusetts; 3 Hunter College, City University of New York, New York, New York; 4 Public Citizen Health Research Group, Washington, DC; 5 Planned Parenthood of Montana, Billings; 6 Middlebury College, Middlebury, Vermont; 7 Harvard T.H. Chan School of Public Health, Boston, Massachusetts

## Abstract

**Importance:**

Several recent US Supreme Court rulings have drawn criticism from the medical community, but their health consequences have not been quantitatively evaluated.

**Objective:**

To model health outcomes associated with 3 Supreme Court rulings in 2022 that invalidated workplace COVID-19 vaccine or mask-and-test requirements, voided state handgun-carry restrictions, and revoked the constitutional right to abortion.

**Design, Setting, and Participants:**

This decision analytical modeling study estimated outcomes associated with 3 Supreme Court rulings in 2022: (1) *National Federation of Independent Business v Department of Labor, Occupational Safety and Health Administration* (*OSHA*), which invalidated COVID-19 workplace protections; (2) *New York State Rifle and Pistol Association Inc v Bruen, Superintendent of New York State Police* (*Bruen*), which voided state laws restricting handgun carry; and (3) *Dobbs v Jackson Women’s Health Organization* (*Dobbs*), which revoked the constitutional right to abortion. Data analysis was performed from July 1, 2022, to April 7, 2023.

**Main Outcomes and Measures:**

For the *OSHA* ruling, multiple data sources were used to calculate deaths attributable to COVID-19 among unvaccinated workers from January 4 to May 28, 2022, and the share of these deaths that would have been prevented by the voided protections. To model the *Bruen* decision, published estimates of the consequences of right-to-carry laws were applied to 2020 firearm-related deaths (and injuries) in 7 affected jurisdictions. For the *Dobbs* ruling, the model assessed unwanted pregnancy continuations, resulting from the change in distance to the closest abortion facility, and then excess deaths (and peripartum complications) from forcing these unwanted pregnancies to term.

**Results:**

The decision model projected that the *OSHA* decision was associated with 1402 additional COVID-19 deaths (and 22 830 hospitalizations) in early 2022. In addition, the model projected that 152 additional firearm-related deaths (and 377 nonfatal injuries) annually will result from the *Bruen* decision. Finally, the model projected that 30 440 fewer abortions will occur annually due to current abortion bans stemming from *Dobbs*, with 76 612 fewer abortions if states at high risk for such bans also were to ban the procedure; these bans will be associated with an estimated 6 to 15 additional pregnancy-related deaths each year, respectively, and hundreds of additional cases of peripartum morbidity.

**Conclusions and Relevance:**

These findings suggest that outcomes from 3 Supreme Court decisions in 2022 could lead to substantial harms to public health, including nearly 3000 excess deaths (and possibly many more) over a decade.

## Key Points

**Question:**

What are the probable health consequences of 3 US Supreme Court decisions in 2022 that invalidated COVID-19 workplace protections, voided state laws on handgun-carry restrictions, and revoked the constitutional right to abortion?

**Findings:**

In this decision analytical modeling study, the model projected that the Supreme Court ruling to invalidate COVID-19 workplace protections was associated with 1402 deaths in early 2022. The model also projected that the court’s decision to end handgun-carry restrictions will result in 152 additional firearm-related deaths annually, and that its decision to revoke the constitutional right to abortion will result in 6 to 15 deaths and hundreds of cases of peripartum morbidity each year.

**Meaning:**

These findings suggest that over a decade, the 3 Supreme Court decisions examined here will contribute to the loss of nearly 3000 US lives and possibly many more.

## Teaser

This decision analytical modeling study evaluates potential public health outcomes associated with 3 Supreme Court decisions in 2022 on COVID-19 workplace protections, handgun-carry restrictions, and constitutional abortion rights.

## Introduction

The US Supreme Court has issued myriad rulings with ramifications for public health and medical care,^1,2^ but few have drawn such widespread criticism, including from the medical community,^3,4,5^ or carried such large potential consequences for the health of US citizens health as 3 landmark cases that it decided in 2022. The first ruling, issued in January 2022 in *National Federation of Independent Business v Department of Labor, Occupational Safety and Health Administration* (*OSHA*),^6^ overturned the OSHA emergency temporary standard (ETS) that would have required large employers to implement measures to protect their workers from COVID-19. Subsequently, over the course of 8 days in June 2022, the court issued several decisions with major public health consequences. In *New York State Rifle and Pistol Association Inc v Bruen, Superintendent of New York State Police* (*Bruen*),^7^ the court decision voided laws in 6 states and the District of Columbia restricting the right to carry handguns, a decision likely to increase handgun carrying and possibly undermine other gun regulations.^5,8,9,10,11^ In *Dobbs v Jackson Women’s Health Organization* (*Dobbs*),^12^ the court overturned *Roe v Wade*, which had protected abortion rights since 1973; abortion bans went into effect immediately in several states and more are forthcoming.

Many commentators have criticized these decisions as a threat to public health.^3,10,13,14^ However, none have comprehensively modeled their quantitative impact. We projected the outcomes of these decisions on mortality and other adverse health consequences.

## Methods

This decision analytical modeling study relied on deidentified publicly available data and was hence deemed not to be human participant research by the Cambridge Health Alliance Institutional Review Board. We summarize our approach to modeling each decision next, and we present detailed methods in the eMethods in Supplement 1. This study adhered to the Consolidated Health Economic Evaluation Reporting Standards (CHEERS) for evaluations of health interventions.

### *OSHA* Decision

On November 5, 2021, OSHA issued the COVID-19 ETS applicable to businesses employing 100 or more workers, with some exclusions.^15^ This standard would have obligated employers to implement a requirement that employees either be vaccinated against COVID-19 or they had to mask and test. Robust evidence supports the efficacy of vaccine mandates in improving vaccine uptake,^16,17,18^ and growing evidence supports the association of mandates with respiratory virus outcomes, including from COVID-19.^19^ The ETS would have been effective on January 4, 2022, near the peak of the first Omicron surge, but was immediately suspended by a federal district court. It was briefly reinstated, before the Supreme Court fully overturned it on January 13, 2022.

We modeled likely health outcomes of the Supreme Court *OSHA* decision invalidating the ETS by building on OSHA estimates of the number of workers who would have been vaccinated as a result of full ETS implementation (18.9 million).^20^ We modeled 3 scenarios that assumed different dates of implementation: for a lower-bound estimate, we assumed the ETS would be effective by the week ending February 5, 2022; for an upper-bound scenario, we assumed it would have been effective by the week ending January 8, 2022; and for our middle (ie, primary) scenario, we assumed that about half of the ETS impact would be realized in mid-January and the full impact by February 2022.

To model deaths, we analyzed several sources. First, using 2022 US Centers for Disease Control and Prevention (CDC) data on COVID-19 deaths by vaccination status, we aggregated deaths by week, vaccination status, and age group (18-29, 30-49, and 50-64 years).^21^ Because these CDC data were only representative of 71% of the US population, we applied the age-group and week-level ratio of unvaccinated deaths to vaccinated deaths from these calculations to the corresponding age-group and week-level death-certificate–based COVID-19 death counts from the National Center for Health Statistics.^22^ These calculations produced our week-level national estimates of deaths among the unvaccinated adult population aged 18 to 64 years.

Next, we analyzed day-level vaccination data from the CDC^23^ and calculated the proportion of all unvaccinated individuals aged 18 to 64 years who would have been vaccinated had the OSHA ETS been implemented for each week. We multiplied this weekly percentage by the weekly death count among those aged 18 to 64 years who were unvaccinated and deflated this by 15% (assuming 85% vaccine efficacy against death)^24^ to arrive at an estimate of the total number of additional weekly deaths due to the Supreme Court *OSHA* decision. We did not attempt to model the impact of mask-and-test requirements for workers who were not vaccinated, given uncertainties of the effect size of these interventions.^25^ Finally, we modeled hospitalizations, intensive care unit (ICU) hospitalizations, and mechanical ventilation hospitalizations by applying the month-specific ratio of deaths to each of these 3 hospitalization outcomes drawn from COVID-19–Associated Hospitalization Surveillance Network data^26^ to our estimates of COVID-19 deaths averted.

### *Bruen* Decision

In the *Bruen* decision, the Supreme Court ruled that the may-issue handgun concealed-carry permit regulations of 6 named states (California, Hawaii, Maryland, Massachusetts, New York, and New Jersey) and the District of Columbia were unconstitutional. These may-issue provisions gave law enforcement discretion in restricting handgun concealed-carry permits; the decision is widely expected to boost the carrying of firearms outside the home, creating a shall-issue or right-to-carry standard in these jurisdictions.^9,27^

The actions of states in response to *Bruen* could attenuate its impact (eTable 1 in Supplement 1 reviews early responses by states), although it is uncertain whether such actions will withstand further legal challenge and some have already been invalidated.^28^ Hence, we modeled 3 post-*Bruen* scenarios. First, for our lower-bound estimate, we projected that actions by states in response to *Bruen* would entirely offset the impact of the decision. Second, we assumed that states were unable to offset any impact of the *Bruen* decision (our upper-bound estimate). For a middle scenario, we assumed that state policy would partially offset *Bruen*, and we projected half the expected outcome (described next) from the implementation of right-to-carry laws in each state. We did not attempt to model expected spillover effects on other states^29^ or the impact of any broader legal shifts signaled by the decision; consequently, each scenario produced conservative estimates of likely health consequences.

Next, we estimated the expected mortality outcome of a shall-issue (ie, nondiscretionary) standard (on any jurisdiction forced to adopt such a standard); eTable 2 in Supplement 1 summarizes contemporary evidence on this issue. Based on the range of outcome estimates from these studies, we projected a lower-bound expected outcome of a 3% increase in firearm-related homicides and an upper-bound outcome of a 9% increase. For a middle estimate, we used a 6% increase.

We then estimated the impact of *Bruen* on firearm-related mortality by applying (ie, multiplying) each of the 3 state policy scenarios to each of the 3 expected outcomes (eTable 3 in Supplement 1), which resulted in a range of outcomes from a 0% to 9% increase; we used the midpoint of this range (4.5%) as our primary estimate. We applied these relative increases to 2020 firearm-related homicides in each state extracted from the CDC WONDER (Wide-Ranging Online Data for Epidemiologic Research) Underlying Cause of Death database. Finally, we projected the number of nonfatal firearm-related injuries by applying an estimate of the ratio of nonfatal firearm-related injuries to firearm-related deaths^30^ to our estimates; we also estimated the number of additional serious injuries by body region.^30^

### *Dobbs* Decision

To estimate the number of deaths associated with mandatory continuation of pregnancies, we first projected the number of foregone abortions consequent to the *Dobbs* decision, which overturned the constitutional right to abortion. Our methodology followed previous work by Myers et al,^31^ who projected foregone abortions in a post-*Roe* nation, updated for this study. In brief, Myers et al (1) used a national data set of abortion facilities, (2) calculated driving time from the centroid of each US county to the nearest abortion facility with and without state abortion bans, and (3) used the causal estimates from Lindo et al^32^ of abortions foregone as a function of driving distance. We mostly followed this approach for our study, with 3 major modifications. First, the directory of abortion facilities was updated to use the Myers Abortion Facility Database,^33^ providing exact dates of operation for all US abortion facilities open at any point between May 1, 2022, and March 30, 2023. Second, we used the most current available estimates of the impact of driving distance from Myers.^34^ Third, we assumed a different set of state abortion bans, under 2 scenarios. In the first scenario, we only assumed that abortion facilities would close in the 13 states enforcing bans on abortion from the moment of conception as of March 30, 2023, as per the Myers database.^33^ In the second, we assumed abortion facility closures in 10 additional states that the Guttmacher Institute^35^ judged likely to ban abortion (referred to hereinafter as high-risk states).

Next, we followed the method of Stevenson,^36^ who calculated the impact of a total national abortion ban on additional pregnancy-related mortality. We assumed, following Stevenson, that each foregone abortion would be associated with an additional 0.8 births in the first full year following the ban, and then multiplied the number of increased births in each state by the state-specific, pregnancy-related mortality rate.^37^ Again following Stevenson,^36^ we subtracted the expected number of abortion-related deaths from this estimate. To project increases in postpartum morbidity among patients forced to continue unwanted pregnancies, we assumed a postpartum hemorrhage rate of 3.21%^38^ and applied this to our estimate of unwanted births.

Finally, we examined safety implications of changed obstetrical management associated with abortion bans, which may put facilities at risk for loss of their licenses or even felony charges. Numerous news reports have described changes in the management of dangerous pregnancy-related complications such as ectopic pregnancy, previable premature rupture of membranes (PPROM), and preeclampsia as consequences of the new state abortion bans. We focused on the impact of 1 complication, previable PPROM (or PPROM near the limits of viability), for which changed management as a result of abortion bans was documented in a recent study.^39^ While patients with previable or periviable PPROM are typically offered 2 options, either termination of pregnancy or expectant (“wait and see”) management, a large academic medical center offered patients only expectant management after implementation of the Texas 6-week abortion ban and noted high rates of maternal morbidity.^39^ Moreover, a recent multicenter study by Sklar et al^40^ found higher rates of morbidity among pregnant patients with periviable PPROM managed expectantly vs with immediate termination of pregnancy. We used these estimates to project the impact of *Dobbs* on peripartum morbidity and on postpartum hemorrhage related to a shift to initial universal expectant management in abortion ban states.

### Statistical Analysis

Data management and analyses were conducted in Stata/SE, version 17 (StataCorp LLC), or Excel, version 16.43 (Microsoft Corp). Detailed statistical methods used in the calculation of foregone abortions were described briefly earlier, and further details are provided in the eMethods in Supplement 1 and Myers et al.^31^ We did not perform statistical tests for hypothesis testing in this modeling study. Data analysis was performed from July 1, 2022, to April 7, 2023.

## Results

### *OSHA* Ruling

The Figure provides weekly trends in deaths among nonvaccinated adults aged 18 to 64 years through May 28, 2022, both without the ETS and assuming the ETS had been implemented according to our primary scenario. The eFigure in Supplement 1 presents a similar figure including all 3 ETS scenarios. Table 1 presents estimates of excess morbidity and mortality summed over the period from January 4 through May 28, 2022. Overall, of an estimated 12 752 deaths among unvaccinated adults aged 18 to 64 years during that period (eTable 4 in Supplement 1), we projected that implementation of the ETS would have averted 1402 deaths in our primary scenario (with lower and upper bounds of 980 and 2940 deaths, respectively).

**Figure.**
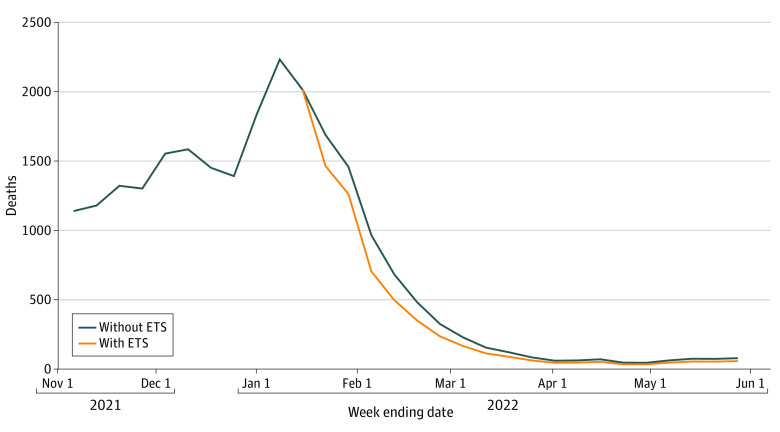
Estimated Weekly Deaths Attributable to COVID-19 Among Nonvaccinated Adults Aged 18 to 64 Years With and Without Implementation of the Occupational Safety and Health Administration Emergency Temporary Standard (ETS) in 2022, Primary Scenario

**Table 1.** Estimated Adverse Health Outcomes Potentially Averted With the US Occupational Safety and Health Administration Emergency Temporary Standard Between January and May 2022

	No. of adverse health outcomes averted		
	Lower bound	Primary scenario	Upper bound
Death	980	1402	2940
Hospitalization	15 793	22 830	48 470
Intensive care unit hospitalization	2641	3739	7738
Mechanical ventilation hospitalization	1042	1472	3036

Table 1 also presents hospitalizations, ICU hospitalizations, and mechanical ventilation hospitalizations averted. Under our primary scenario, implementation of the OSHA ETS would have averted 22 830 hospitalizations, 3739 ICU stays, and 1472 stays involving mechanical ventilation through May 28, 2022.

### *Bruen* Ruling

Table 2 presents estimates of increases in firearm-related mortality and nonfatal injuries in each state in each of our 3 main scenarios (eTable 5 in Supplement 1 provides death estimates for additional intermediate scenarios). For our primary scenario, we projected 152 additional firearm-related deaths annually (with lower and upper bounds of 0 and 304 deaths, respectively). Our upper-bound estimate projected state-level mortality increases ranging from 1 death in Hawaii to 156 in California. We also projected 377 additional nonfatal firearm-related injuries (with lower and upper bounds of 0 and 754). These injuries include an estimated 29 serious head or neck injuries, 51 serious chest injuries, 37 serious abdominal or pelvic injuries, and 77 serious extremity injuries in our primary scenario (eTable 6 in Supplement 1).

**Table 2.** Expected Annual Increase in Number of Firearm-Related Homicides in 6 May-Issue States and the District of Columbia as a Result of *Bruen*

State	No. of firearm-related homicides in 2020^a^	Increase in No. of firearm-related deaths^b^			Increase in No. of firearm-related nonfatal injuries^b^		
		Lower bound	Primary scenario	Upper bound	Lower bound	Primary scenario	Upper bound
California	1732	0	78	156	0	193	387
Hawaii	16	0	1	1	0	2	4
Maryland	526	0	24	47	0	59	117
Massachusetts	130	0	6	12	0	15	29
New York	561	0	25	50	0	63	125
New Jersey	253	0	11	23	0	28	56
District of Columbia	157	0	7	14	0	18	35
Total	3375	0	152	304	0	377	754

^a^ Data are from the US Centers for Disease Control and Prevention WONDER (Wide-Ranging Online Data for Epidemiologic Research) Underlying Cause of Death file.

^b^ Increases in firearm-related deaths and firearm-related nonfatal injuries are presented with lower bound (equal to a 0% change), primary scenario (equal to a 4.5% increase), and upper bound (equal to a 9% increase) values. Values may not precisely sum to totals due to rounding.

### *Dobbs* Ruling

We estimated that abortion bans would be associated with 30 440 fewer abortions annually in the 13 states that had such bans as of March 30, 2023, related to increased driving distance to the nearest abortion facility (Table 3). Under a scenario in which other, high-risk states also enacted abortion bans, we projected 76 612 fewer abortions.

**Table 3.** Expected Annual Increase in Pregnancy-Related Mortality and Morbidity Due to Foregone Abortions as a Result of *Dobbs*

	No. of adverse health outcomes resulting from abortion bans	
	In states with current bans only	In states with current bans plus in high-risk states
Foregone abortions	30 440	76 612
Increase in pregnancy-related deaths	6	15
Increase in peripartum hemorrhage	782	1967

Due to these foregone abortions, we projected an estimated 6 additional pregnancy-related deaths annually in our first scenario and 15 additional yearly deaths if all high-risk states were also to ban abortion (Table 3). We also projected an additional 782 and 1967 cases of postpartum hemorrhage in these 2 scenarios, respectively.

Finally, we projected an additional 454 cases of peripartum morbidity and 202 cases of hemorrhage associated with changed management of PPROM in our first scenario; we projected 826 and 367 cases, respectively, in a scenario where all high-risk states ban abortion (eTable 7 in Supplement 1).

## Discussion

In this decision analytical modeling study, we estimated (in our primary scenarios) that the Supreme Court *OSHA* decision invalidating workplace protections was associated with 1402 additional COVID-19 deaths among workers aged 18 to 64 years in early 2022. We also estimated that *Bruen*, the court’s expansion of handgun carry, will be associated with approximately 152 deaths annually. Finally, we estimated that the *Dobbs* nullification of *Roe v Wade* will be associated with 6 to 15 pregnancy-related deaths annually. Together, our findings suggest that these 3 decisions may contribute to nearly 3000 additional deaths over the course of a decade (2022-2031), assuming no outcomes from the *OSHA* ruling after May 2022 and no outcomes from *Bruen* or *Dobbs* in 2022 with stable annual impacts thereafter; that toll would be about 2-fold higher under our high-end, perhaps more realistic assumptions. We also projected approximately 22 830 additional COVID-19 hospitalizations in 2022, as well as 377 additional nonfatal firearm-related injuries, 982 additional cases of postpartum hemorrhage, and 454 cases of maternal morbidity from changes in obstetrical management annually. Given the populations most affected by these decisions (eg, workers aged 18-64 years or women of reproductive age), it is probable that the majority of these excess deaths, injuries, and complications will occur among working-age adults and adolescents.

Previous studies have examined the impact of vaccination on population-wide COVID-19 mortality^41^ as well as the effectiveness of mandates on vaccine uptake in different settings.^18,19,42^ A previous study by OSHA^20^ specifically projected outcomes of the proposed ETS on COVID-19–related illness and deaths. Our estimate of 1402 deaths preventable by ETS implementation was substantially lower than the OSHA estimate of 6830 deaths prevented, reflecting our more conservative methods and assumptions. For instance, our primary scenario assumed a lag in outcomes attributable to the ETS owing to decisions by lower courts before the Supreme Court accepted the case. Had we assumed that the ETS was implemented on January 2, 2022, our primary estimate would be closer to the OSHA estimate. Moreover, neither our analysis nor the OSHA primary estimate included potential outcomes of reduced transmission (ie, to family members); a recent analysis examining outcomes of college-level vaccine mandates suggested that such spillover benefits may be substantial.^19^ Hence, both estimates should be regarded as conservative.

Our projections of additional firearm-related homicides (and injuries) associated with the *Bruen* decision were lower than what would be expected from a more comprehensive reversal of gun regulations.^43^ However, *Bruen* invalidated only particular regulations (and in only 7 jurisdictions) expanding the Supreme Court’s previous decisions that had already led to substantial firearm deregulation (eg, *District of Columbia v Heller*^44^). Still, the impact of *Bruen*, as some legal scholars have noted, might be broader; it rests on a novel argument (ie, a requirement for historical precedents for contemporary regulations) that the court may use to further prioritize gun rights over health outcomes.^5^ Although *Bruen* may ultimately “reverberate much more widely”^8^ and be used by lower courts to invalidate other gun regulations, we modeled only its impacts on right-to-carry policies in 7 jurisdictions. Finally, while it is conceivable that a reduction in firearm-related homicides might result in “substitution” with homicide via other means, recent research suggests that this outcome is negligible or nonexistent.^45^

Stevenson^36^ previously estimated mortality associated with the repeal of *Roe v Wade*, projecting 140 additional pregnancy-related deaths in the first full year following a national abortion ban. Our approach emulated some of Stevenson’s methods but posited bans in some, rather than all, states and estimated the number of foregone abortions in those states as a function of changing travel time to abortion facilities. Consequently, our modeling accounted for the fact that some individuals will obtain abortions out of state. However, we elected not to model the effects of congestion at abortion facilities in states without bans, which will likely further delay and reduce abortions (and worsen health outcomes) until facilities are able to expand capacity. In addition to pregnancy-related mortality, we examined maternal morbidity (specifically postpartum hemorrhage) as well as health outcomes attributable to changed obstetrical management of PPROM due to abortion bans. However, our analysis did not address delays due to *Dobbs* in receipt of abortion. Such delays are likely to increase the proportion of abortions performed in the second trimester, which is associated with more complications (including hemorrhage) relative to earlier abortions. Additionally, we elected not to model the impact of the likely increase in self-managed abortion^46^ or worsened management of ectopic pregnancy, previable preeclampsia, or miscarriage, given greater uncertainty for these outcomes. Abortion denial is also expected to force some individuals (and their family members) into poverty,^47^ an important social determinant of ill health that we also did not model. These are important areas for future research.

### Limitations

This decision analytical modeling study has some additional limitations. Modeling required consideration of studies that often provided a range of plausible effect estimates for a particular policy (eg, of right-to-carry laws). Moreover, health outcomes of each Supreme Court decision will be shaped by future executive branch rulemaking, legislative actions, and court decisions as well as other behavioral impacts (eg, decisions on voluntary vaccine mandates imposed by businesses, use of self-managed medication abortion, or state firearm culture). Given this complexity, together with variability in the underlying studies we used to estimate policy impacts, we eschewed probabilistic modeling and instead modeled a range of scenarios for each decision, while hewing to an overall conservative approach. Finally, our modeling pertained only to the 3 decisions described and does not speak to health outcomes associated with the Supreme Court’s other 2022 decisions.

## Conclusions

In this decision analytical model study, we estimate that over a decade, nearly 3000 lives (and likely many more) will be lost due to 3 Supreme Court decisions in 2022. Its conservative reshaping in recent years has yielded decisions with substantial impacts on many aspects of life in the US, including health, as we examine here. The findings of this study suggest that these Supreme Court decisions may harm the health of US citizens for years, and possibly decades, to come.

## Notes

Supplement 1. **eMethods.** Supreme Court Rulings Examined in This Study and Approach to Modeling **eFigure.** Deaths Among Nonvaccinated Adults Aged 18 to 64 Years With and Without Implementation of the Occupational Safety and Health Administration Emergency Temporary Standard (ETS) in 3 Scenarios **eTable 1.** Policy Responses of May-Issue [Firearm Carry License] States Following the *Bruen* Decision **eTable 2.** Studies Examining Impacts of Shall-Issue [License to Carry] (or Related) Right-to-Carry State Provisions **eTable 3.** Effect of *Bruen* on State Firearm Death Rates Under Differing State Policy Responses and Assumptions About Right-to-Carry Law Effects **eTable 4.** Estimated Effects of Implementation of the Occupational Safety and Health Administration Emergency Temporary Standard (ETS) Midpoint Estimate (Primary Scenario) **eTable 5.** Annual Increase in the Number of Firearm-Related Homicides in 6 May-Issue States as a Result of *Bruen*, All Scenarios **eTable 6.** Annual Increase in the Number of Firearm-Related Nonfatal Injuries Resulting in Significant Body Region Injury **eTable 7.** Annual Increase in Morbidity Due to Changed Management of Previable Premature Rupture of Membranes as a Result of *Dobbs v Jackson Women’s Health Organization*
eReferences

Supplement 2.
Data Sharing Statement
