# Supplementary material for: Projected Health Outcomes Associated With 3 US Supreme Court Decisions in 2022 on COVID-19 Workplace Protections, Handgun-Carry Restrictions, and Abortion Rights
Source: JAMA Netw Open. 2023 Jun 8;6(6):e2315578. doi: 10.1001/jamanetworkopen.2023.15578 (PMC10251209; doi:10.1001/jamanetworkopen.2023.15578)
Supplement: Supplement 1. — eMethods. Supreme Court Rulings Examined in This Study and Approach to Modeling eFigure. Deaths Among Nonvaccinated Adults Aged 18 to 64 Years With and Without Implementation of the Occupational Safety and Health Administration Emergency Temporary Standard (ETS) in 3 Scenarios eTable 1. Policy Responses of May-Issue [Firearm Carry License] States Following the Bruen Decision eTable 2. Studies Examining Impacts of Shall-Issue [License to Carry] (or Related) Right-to-Carry State Provisions eTable 3. Effect of Bruen on State Firearm Death Rates Under Differing State Policy Responses and Assumptions About Right-to-Carry Law Effects eTable 4. Estimated Effects of Implementation of the Occupational Safety and Health Administration Emergency Temporary Standard (ETS) Midpoint Estimate (Primary Scenario) eTable 5. Annual Increase in the Number of Firearm-Related Homicides in 6 May-Issue States as a Result of Bruen, All Scenarios eTable 6. Annual Increase in the Number of Firearm-Related Nonfatal Injuries Resulting in Significant Body Region Injury eTable 7. Annual Increase in Morbidity Due to Changed Management of Previable Premature Rupture of Membranes as a Result of Dobbs v Jackson Women’s Health Organization eReferences [file jamanetwopen-e2315578-s001.pdf]

Supplementary Online Content

Gaffney A, Himmelstein DU, Dickman S, et al. Projected health outcomes associated with 3 US Supreme Court decisions in 2022 on COVID-19 workplace protections, handgun-carry restrictions, and abortion rights. *JAMA Netw Open*. 2023;6(6):e2315578. doi:10.1001/jamanetworkopen.2023.15578

eMethods. Supreme Court Rulings Examined in This Study and Approach to Modeling

eFigure. Deaths Among Nonvaccinated Adults Aged 18 to 64 Years With and Without Implementation of the Occupational Safety and Health Administration Emergency Temporary Standard (ETS) in 3 Scenarios

eTable 1. Policy Responses of May-Issue [Firearm Carry License] States Following the *Bruen* Decision

eTable 2. Studies Examining Impacts of Shall-Issue [License to Carry] (or Related) Right-to-Carry State Provisions

eTable 3. Effect of *Bruen* on State Firearm Death Rates Under Differing State Policy Responses and Assumptions About Right-to-Carry Law Effects

eTable 4. Estimated Effects of Implementation of the Occupational Safety and Health Administration Emergency Temporary Standard (ETS) Midpoint Estimate (Primary Scenario)

eTable 5. Annual Increase in the Number of Firearm-Related Homicides in 6 May-Issue States as a Result of *Bruen*, All Scenarios

eTable 6. Annual Increase in the Number of Firearm-Related Nonfatal Injuries Resulting in Significant Body Region Injury

eTable 7. Annual Increase in Morbidity Due to Changed Management of Previable Premature Rupture of Membranes as a Result of *Dobbs v Jackson Women's Health Organization*

eReferences

This supplementary material has been provided by the authors to give readers additional information about their work.

eMethods. Supreme Court Rulings Examined in This Study and Approach to Modeling

National Federation of Independent Business v. OSHA

I. *Background and Timeline*

OSHA issued a Vaccination and Testing Emergency Temporary Standard (ETS) on November 5, 2021.[1] The ETS applied to entities (businesses, non-profits entities, and some governmental agencies) employing 100 workers or more, excluding employees who worked (1) only outdoors; (2) only at home; (3) or in places where no other individuals were present.[1] It required covered employers to "develop, implement, and enforce a mandatory COVID–19 vaccination policy, with an exception for employers that instead adopt a policy requiring employees to either get vaccinated or to elect to undergo regular COVID–19 testing and wear a face covering at work in lieu of vaccination."[1] The effective date was set at January 4, 2022, with a requirement that unvaccinated workers begin masking on December 5.[2]

Immediately after issuance of the ETS, a coalition of states, businesses and other groups filed a petition with the Fifth Circuit US Court of Appeals contending that the rule was unconstitutional, and on November 6 a panel of judges issued a temporary injunction barring enforcement of the rule.[2] The Biden Administration defended the standard in a filing before the court the following Monday,[3] calling for a withdrawal of the court's initial ruling, but on November 12 the Fifth Circuit issued a ruling extending the stay on implementation of the ETS.[4] Meanwhile, other lawsuits had been filed in courts across the country, and 34 of them were consolidated into a single case and randomly assigned to the Sixth Circuit federal appeals court in Cincinnati.[5] On December 17, the Sixth Circuit overturned the Fifth Circuit's decision, effectively reinstating the ETS.[6] The case was

© 2023 Gaffney A et al. *JAMA Network Open*.

immediately appealed, however, to the Supreme Court, and on December 22, the Supreme Court announced that it would decide the case as part of its "shadow docket." On January 7, it decided that OSHA had overstepped its legal authority and voided the ETS.

II. Number of workers who would have been vaccinated under the ETS

We estimated deaths stemming from the Supreme Court's voiding of the ETS by building off of OSHA's 2021 published estimates of the number of people who would have been vaccinated had the ETS remained in force.[7] OSHA also projected health impacts of the ETS for the period April – August 2021, including deaths averted.[7] Our analysis differs from OSHA's in using the actual number of COVID-19 deaths and vaccination rates that occurred in the wake of the January 7, 2022 ruling, whereas OSHA's projections were based on previous months deaths. We also adjusted OSHA's methods to account for delays in implementation of the rule attributable to the lower court decisions.

The starting point for our analysis is OSHA's estimate that 18.9 million workers would have been vaccinated because of the ETS. OSHA used multiple sources to calculate the number of workers who would fall within the parameters of the ETS, excluding, for instance, those expected to exclusively telework.[1] It estimated that the ETS would cover 263,879 entities employing 102.7 million employees, 84.2 million of whom would be subject to the ETS and not exclusively teleworking or working outdoors (Table IV.B.5.).[1] It then estimated the vaccination rate of these workers using age-specific data from October 4, 2021 drawn from the CDC COVID Data Tracker (it estimated that 38.7% of covered workers, or 31.7 million, were unvaccinated). It then adjusted this figure for ongoing trends in vaccination, classifying the currently unvaccinated as either vaccine-hesitant or not-hesitant using survey data. It then classified the proportion of the vaccine-hesitant

© 2023 Gaffney A et al. *JAMA Network Open*.

who would likely receive a medical or religious exemption. Finally, it estimated the number of otherwise unvaccinated workers likely to be vaccinated under the ETS, via two mechanisms: (1) implementation of a voluntary employer mandate due to the ETS, or (2) the ETS mandate itself. For the first, it assumed that the proportion of employers with voluntary vaccine mandates would rise from 25% (at baseline) to 60% with the ETS. Overall, it estimated that 22,751,767 individuals would be vaccinated because of the ETS (Table IV.B.8.).

However, that figure included 1.9 million workers in healthcare settings, who were excluded by OSHA for the purposes of its health impacts analysis, leaving 20.8 million workers who would have gotten vaccinated as a result of the ETS.[8] In its health impacts analysis, OSHA further downwardly adjusted this figure to 18.9 million workers after excluding workers covered by federal Contractor Guidance and those ages 65-74.[7] We use this figure, 18,914,528, as the starting point for our estimates of workers who would have been vaccinated had the Supreme Court not voided the ETS.

III. Effective Date of the ETS

The ETS would have gone into effect on January 4, 2022 had the Supreme Court not invalidated it. However, timing the impact of the OSHA rule on vaccination rates had the Supreme Court acted differently is not straightforward. Had there been no uncertainty at all about the future of the ETS (and no stay from the Fifth Circuit), the impact of the vaccine mandates would probably have begun to be realized after issuance of the rule in early November, as employers pushed to comply with the mandates in advance of the January 4 deadline. In this scenario, we would expect some COVID deaths to be averted in November and December of 2021.

© 2023 Gaffney A et al. *JAMA Network Open*.

However, the Fifth Circuit's initial stay probably attenuated early compliance with the ETS, likely negating some or all of its effects on COVID death rates even prior to the Supreme Court's January 7 decision. Moreover, even if, on December 22, 2021 the Supreme Court had declined to consider reversing the Sixth Circuit's reinstatement of the ETS, employers and employees would have had only 2 weeks to meet the January 4 mandate deadline, suggesting that the "full effect" of the mandate would not have been realized during all of January.

Given these complexities, we modelled three scenarios. For our "lower bound" estimate, we assume that almost no deaths would have been averted by the ETS during January, because of the uncertainty created by the lower court ruling (specifically, we assume that the health effects of the ETS would not have been felt prior to the week ending February 5, 2022). In our "upper bound" scenario, we assume that had the Supreme Court not agreed on December 22 to hear the case, implementation of the ETS vaccine mandate together with masking requirements and testing requirements for unvaccinated workers would have been effectively implemented throughout January 2022 (i.e. full effect of the ETS realized as of the week ending in January 8, 2022). For our mid-point (primary) estimate, we assume that approximately half of the ETS' effect on vaccination rates would have been realized in January (i.e for the weeks ending January 22 and 29, 2022), and the full effect by the week ending February 5, 2022.

IV. *Modelling deaths*

We based our estimates of the number of deaths potentially avertable by implementation of the ETS by analyzing two data sources on COVID-19-related deaths.

© 2023 Gaffney A et al. *JAMA Network Open*.

We first analyzed week-level data on death counts by vaccination status provided by the CDC. These data reflect deaths that occurred through May 28, 2022 and were obtained on August 8, 2022.[9] In these data, vaccinated cases (as opposed to unvaccinated cases) are those among persons who tested positive for COVID-19 via a SARS-CoV-2 RNA or antigen test that was performed 14 days or more after "verifiably completing the primary series of an FDA-authorized or approved COVID-19 vaccine." COVID-19 deaths are those that occur "in a person with a documented COVID-19 diagnosis who died"; death dates reflect when the individual tested positive for COVID-19 as opposed to the day of death. These data was collected from 31 health departments that collectively represent 71% of the total US population (at the time of data download); data on death were provided by all but one of these departments. We calculated weekly deaths among unvaccinated individuals in three age groups: 18-29, 30-49, and 50-64 age groups. We then calculated the age-group and week specific proportion of unvaccinated COVID deaths over total COVID deaths. However, this data, as mentioned, was not nationally-representative. To estimate the total number of deaths among the unvaccinated ages 18-64, we then analyzed week-level death certificate-based data from the National Center for Health Statistics (NCHS) on COVID-19 related deaths (ICD-10 code U07.1),[10] and applied the CDC-data derived age-group and week-specific ratio of unvaccinated / total COVID deaths to the corresponding age-group and week-specific NCHS COVID death figures.

Next, we calculated the number of these deaths that would have been averted through implementation of the ETS as follows. For each week, we calculated the share of total unvaccinated individuals ages 18-64 who *would* have been vaccinated under the ETS. Data on the number of individuals 18-64 who were fully vaccinated at the end of each week was calculated by analyzing day-level vaccine data provided by the CDC[11]; estimates for the number of unvaccinated individuals was

© 2023 Gaffney A et al. *JAMA Network Open*.

calculated by subtracting these figures from the 2021 total working age (18-64 years) population per the Census Bureau.[12] As previously noted, we then assumed that all 18,914,528 individuals that OSHA estimated would be vaccinated as a result of the ETS were vaccinated by the first week of January for scenario 1, and by the first week of February for scenario 2. For scenario 3 (our midpoint scenario) we assumed that half the expected vaccinations would have taken place by weeks 3-4 of January, and all by the first week of February (week ending February 5, 2022). We then applied the percentage of non-elderly unvaccinated adults who would have been vaccinated under the ETS for each week to the weekly unvaccinated non-elderly adult death count. Finally, we multiplied this figure by .85% under the assumption that vaccination would reduce the risk of death by 85%.[13]

These calculations can be more clearly appreciated in Appendix Table 1, with provides details of the estimates of our primary scenario. For instance, for the week ending January 22, 2022, there were 60.2 million unvaccinated non-elderly adults in the US (Column B). We assumed that 9.5 million of these (half of OSHA's estimated 18.9 million vaccinated under the ETS) would have been vaccinated due to the ETS at that point (Column C), equivalent to 15.7% of all unvaccinated non-elderly persons (Column D). That week, an estimated 1,688 deaths occurred among unvaccinated individuals ages 18-64 (Column E). We hence assumed that 15.7% of these deaths (265) would have been averted assuming 100% vaccine efficacy against death (Column F), which we then deflated to 266 assuming 85% vaccine efficacy (Column G). This would have reduced the number of deaths among non-vaccinated non-elderly adults to 1,462 that week (Column H). Similar calculations were made for each week and for each of the three scenarios (note: small inconsistencies are due to rounding).

V. *Modeling Hospitalizations*

Finally, we modeled hospitalizations using data from Coronavirus Disease 2019 (COVID-19)-Associated Hospitalization Surveillance Network (COVID-NET), a surveillance system that collects data on hospitalized COVID-19 patients at some 250 hospitals across 14 states.[14] We used month-level COVID-NET aggregate statistics on the proportion of hospitalized COVID-19 patients who died, were in the ICU, and who were mechanically ventilated to calculate month-level estimates of the number of COVID-19 hospitalizations, ICU hospitalizations, and mechanical ventilation hospitalizations per COVID-19 death. We then multiplied these figures by the number of COVID-19 deaths that would have been averted by the ETA under each scenario each week to calculate the number of hospitalization outcomes that would have been averted under the ETS.

© 2023 Gaffney A et al. *JAMA Network Open*.

New York State Rifle and Pistol Association Inc. vs. Bruen

I. *States affected*

In modelling the effects of *New York State Rifle and Pistol Association Inc. vs. Bruen* we only considered jurisdictions explicitly named by the Court as having "may issue" standards (California, Hawaii, Maryland, Massachusetts, New York, New Jersey, and the District of Columbia). However, it has been argued that the court's embrace of a novel legal precedent in *Bruen* — one centered on finding historical precedent for gun regulations and neglecting policy impacts — could have much broader impacts, potentially culminating in the lower courts invalidating numerous gun regulations throughout the nation.[15–17] As one legal scholar put it, the courts' embrace of "a history-only test for all future Second Amendment challenges will reverberate much more widely."[18] Or as legal scholar John Donahue noted,

> *"Bruen has created an unworkable and largely nonsensical standard for evaluating gun regulations based on history when the history has very little to say about wise policy today. Hopefully, the standard will not be used to invalidate important tools to address gun violence, such as state bans on assault weapons and high-capacity magazines, red flag laws, safe storage laws, waiting periods, and other sensible measures designed to reduce the large social costs of gun violence in America. But the standard is so vague and malleable that this Supreme Court will be able to sustain – or strike down – any of these measures and many more."*[19]

Hence, it is possible that the long-term impact of *Bruen* will go well beyond the immediate effect on concealed carry permits in these 7 jurisdictions. However, given the difficulty of making quantitative projections of such a broader (and more uncertain) shift in legal context, we limited our analysis to

© 2023 Gaffney A et al. *JAMA Network Open*.

the direct and narrow impact of the expanded issuance of carry permits in those specific jurisdictions only, erring towards conservative estimates.

II. Policy impacts for affected states

The immediate impacts for firearm policy in affected states will still likely be substantial. As Donahue further argues:

> *"The narrow effect of the decision will be to expand gun carrying outside the home in the relatively limited number of states that have tried to be more restrictive in this dimension … In turning these states into "right-to-carry" states that essentially give anyone (other than a narrow class of prohibited possessors) the right to carry handguns outside the home, the Court determined that its judgment about gun policy trumped the legislative determinations based on strong empirical evidence that promiscuous gun carrying will elevate violent crime."*[19]

However, by most accounts, the impact of this ruling will still be shaped by state policy responses. While the Supreme Court declared subjective "may issue" criteria to be unconstitutional, it left room for states to continue to impose (and potentially expand) objective criteria for the issuance of carry permits. Appendix Table 2 reviews the diverse and conflicting steps taken by state governments in the immediate wake of the *Bruen* decision.

State policy, in other words, could substantively modify the impact of *Bruen*, although a key (and unanswered) question is which of these policies will withstand further legal challenges. For instance, in response *Bruen*, New York State passed legislation that created numerous new restrictions on

© 2023 Gaffney A et al. *JAMA Network Open*.

handgun carry; however, in October, a Federal court declared many of these provisions unconstitutional.[20] Hence, we modelled three different "state policy scenarios" for how the court's decision could affect these jurisdictions.

In the first policy scenario, we assume that the impact of *Bruen* in each jurisdiction will be entirely offset by policies that fully constrain a growth in handgun carry, and that these policies furthermore survive legal challenge. We view this as an unlikely outcome, but provide it as our "lower bound" estimate.

In the second scenario, we assume that *Bruen* instigates statutory change that cannot be offset by other legislation or state policy change (possibly because such policies do not withstand legal challenge). Consequently, in this scenario, each state experiences the full "expected outcome" of right-to-carry (RTC) laws based on the experiences of other states that have implemented such policies in the past. We use this scenario to model our "upper bound" estimate, although it may nevertheless be conservative for two reasons. First, as previously noted, a broader legal shift due to *Bruen* could have larger — although difficult to project — effects nationwide. Second, we did not model potential spillover effects on neighboring states from policy changes in the 7 affected jurisdictions; however, a growing body of research points to such spillovers (e.g. via gun theft) as an important mechanism connecting RTC laws and violent crime.[21]

Finally, for our primary policy scenario, we assume that state policy responses will *partially* offset the impact of *Bruen* on handgun carry. In this scenario, we assume half the "expected outcome" of the implementation of RCT laws in each state.

© 2023 Gaffney A et al. *JAMA Network Open*.

III. Modelling "right to carry" on health ("expected outcomes")

Next, we derived estimates of the "expected outcome" of the implementation of a "shall issue" or RTC legal standard via a review of the literature. Our focus was on more recent (and methodologically stronger) studies. As noted by Donahue et al.,[21] while older studies of the effects of RTC laws had mostly null findings, "the predominant conclusion from studies in the last five years has been that RTC laws increase violent crime." In part that may be because the impact of RTC laws has changed over time; as we are interested in the contemporary (i.e. not historical) effect of these laws, we rely on analyses that use more recent data. However, perhaps more importantly, and as others have noted,[21,22] much of the older literature had serious methodological shortcomings. Key recent national studies from the past 5 years are summarized in Appendix Table 3.

Each of the studies summarized in Appendix Table 3 demonstrates positive associations between RTC laws and firearm deaths and/or violent crime, although not all associations with deaths are significant. Effect estimates for impacts on firearm homicide or overall homicide range from a 3% increase (Schell et al.[22]) to a 9% increase (Siegel et al.).[23] Donhue et al.[21], in a city-based analysis, also identified a 9% increase but standard errors were wide and the estimate was not statistically significant (although violent crime estimates were larger and significant). Crifasi et al.[24] identified a 14% reduction in death with the introduction of permit-to-purchase laws (implying a 16% increase in deaths with their nullification), but these laws are related yet not directly comparable to RTC laws and so we did not use this estimate.

Based on this range of effect estimates, we assumed a lower-bound "expected outcome" estimate of a 3% increase in firearm-related deaths in affected states. For our higher-bound estimate, we

© 2023 Gaffney A et al. *JAMA Network Open*.

assumed a 9% increase. For our mid "expected outcome" estimate, we used the midpoint between these two estimates, or 6%.

Finally, as shown in Appendix Table 4, we then applied (i.e. multiplied the percentages) of each of these three "expected outcome" estimates (3%, 6%, or 9% increases in deaths) to each of the three aforementioned "state policy scenarios" (0%, 50%, or 100% of the "expected outcome"), to produce 6 (unique) changes to firearm firearm-related mortality: 0%, 1.5%, 3%, 4.5%, 6%, and 9% relative increases. We chose the midpoint of this range, 4.5%, as our primary effect estimate.

IV. Estimated additional firearm deaths

We estimated the impact of *Bruen* on firearm deaths by applying each of the 6 unique relative changes in firearm-related mortality to 2020 firearm-related homcide to each of the 6 affected states as well as the District of Colombia, using data on all abstracted from CDC WONDER Underlying Cause of Death data.[25] Although we present all 6 mortality estimates in the appendix (Appendix Table 5), in the manuscript (Table 2) we only present our "low" estimate (0% effect on gun deaths), our middle or primary estimate (a 4.5% increase), and our high estimate (a 9% increase using the highest estimate for each parameter). Each such estimate, however, for reasons reviewed above, should be interpreted as conservative.

V. Estimated additional firearm injuries

To estimate the number of additional firearm-related injuries as a result of *Bruen*, we relied on a recent national study by Kaufman et al.[26] These investigators estimated 2009-2017 firearm deaths by

© 2023 Gaffney A et al. *JAMA Network Open*.

analyzing the CDC WONDER database and firearm-related nonfatal injuries by analyzing the National Emergency Department Sample (NEDS), an administrative database that includes approximately 20% of all US emergency department (ED) visits.[26] They calculated an annual average of 85,694 nonfatal injuries and 34,538 deaths over the study period, giving a nonfatal injury : death ratio of 2.48. We applied this ratio to our estimate of increased deaths to estimate the increase in nonfatal injuries. We additionally use their estimates of rates of what we term "significantly" injured body regions (defined as abbreviated injury score [AIS] of >= 2) among those with nonfatal injuries (results provided in Appendix Table 6).

Dobbs v. Jackson Women's Health Organization

1. *Modelling foregone abortions*

We first projected the number of foregone abortions by state because of the overturning of *Roe v. Wade*. To do so, we used the approach of Myers et al,[27] which we updated for this paper.

In brief, in their previous study, Myers et al.[27] projected the number of foregone abortions post-Roe using a database of abortion providers in the US kept by the Advancing New Standards in Reproductive Health (ANSIRH) at the University of California, San Francisco. To project the effect of facility closure on the number of abortions performed, they relied on causal estimates from Lindo et al.,[28] whose difference-in-difference analysis assessed the impact of abortion clinic closures in Texas on abortion rates as a function of driving time. Myers et al. then estimated the travel time (via car) from the centroid of each US county to the closest abortion clinic, under current law and under

© 2023 Gaffney A et al. *JAMA Network Open*.

different post-Roe scenarios, and calculated how changes in driving time to the nearest abortion clinic would alter the number of abortions performed in each state.

The Myers et al.[27] analysis was revised for the current study, as described in the main manuscript. Revision include: (1) an update of the directory of abortion providers[29]; (2) more recent estimates of the effects of driving distance on abortion provision[30]; and (3) different assumptions about which states would ban abortion. Specifically, we examined foregone abortions under two scenarios. In the first scenario, we only treated those states with abortion bans in effect at the end of March 30 2023 as having abortion bans, per the Myers database.[29] In the second scenario, we projected the impact if states that the Guttmacher Institute considers "likely" to enact abortion bans.[31]

2. *Deaths from Foregone Abortions*

To examine the effect of foregone abortions on maternal mortality, we followed a similar approach as Stevenson et al., who projected additional deaths caused by a total national abortion ban.[32]

We first calculated the state-level maternal mortality rate in 2018-2020 using death-certificate data on state-level maternal mortality for 2018-2020 from the National Center of Health Statistics[33] and data on 2018-2020 births from CDC WONDER.[34] We also estimated the number of maternal deaths by state in 2019 by applying these state-level 2018-2020 rates to the number of births in each state in 2019, also obtained from CDC WONDER.[34]

We drew on these estimates of 2019 state-level maternal mortality rates, as well as the number of abortions performed in each state in 2020 according to a Guttmacher analysis,[35] to calculate the

© 2023 Gaffney A et al. *JAMA Network Open*.

number of maternal deaths from foregone abortions by applying the method of Stevenson.[32] Stevenson assumed that each foregone abortion results in 0.8 additional births (the figure is less than 1 as some pregnancies end in miscarriage), and then multiplied 0.8 * number of abortions denied * pregnancy-related mortality rate per birth. Stevenson subtracted from this figure an estimate of the number of pregnancy-related deaths among patients who obtain abortions (0.7 / 100,000) to estimate additional net deaths due to foregone abortions. Stevenson projected smaller effects for the first year of ban; we chose to model deaths in the first full year following the implementation of a ban, but otherwise used her approach, albeit applying it to our state-level, travel-time based estimates of foregone abortions, described above.

3. *Postpartum Hemorrhage due to Foregone Abortions*

As an indicator of increased morbidity stemming from foregone abortions among those with unwanted pregnancies, we calculated the number of additional cases of postpartum hemorrhage annually. To do so, we relied on a study by Reale et al., who examined trends in postpartum hemorrhage using the National Inpatient Sample, a large all-payer hospital administrative database.[36] Reale et al. estimated a 3.21% postpartum hemorrhage rate among all deliveries in 2014.[36] We applied this rate to the number of foregone abortions (minus 20% to account for miscarriage, following Stevenson[32]) annually.

4. *Peripartum Morbidity due to Changed Obstetrical Management of Previable Premature Rupture of Membranes (PPROM)*

© 2023 Gaffney A et al. *JAMA Network Open*.

Multiple media reports have described harmful alterations in obstetrical care due to abortion bans, including for treatment of ectopic pregnancy, previable premature rupture of membranes (PPROM), and eclampsia.[37–39] This has been attributed to the "chilling effect" of these laws, i.e. physicians' or hospitals' concerns about facing criminal charges from provision of abortion care even when the pregnant patient's life is in danger (which, in most states, theoretically qualifies as an exemption from state abortion bans). Although there is some uncertainty as to how this will impact the care of patients with such conditions in the longer term, we modelled the impact of *Dobbs* for treatment of one condition — previable PPROM (or PPROM at the limits of fetal viability) — where a recent study found evidence of harm from an abortion ban.[40]

That study examined the effect of Texas law SB 8, a 6 week abortion ban, on the management of previable PPROM.[40] Patients with PPROM are usually offered two treatment options initially: termination (via labor induction or dilation and evacuation) or expectant management. However, after the enactment of SB8, women with PPROM were *only* offered expectant management.[40] Many of these women experienced serious maternal morbidity: 36% developed chorioamnionitis, 4% were admitted to the ICU, 18% had postpartum hemorrhage requiring transfusion, and 57% had any maternal morbidity.[40]

A recent four-state study by Sklar et al.[41] directly compared outcomes among pregnant patients with PPROM treated with expectant management vs. termination of pregnancy, and found significantly higher rates of complications with expectant management. Overall, 60.2% of patients treated with expectant management experienced maternal morbidity, approximately twice the proportion treated with termination of pregnancy (33.0%); the corresponding proportions for postpartum hemorrhage were 23.1% and 11.0%.

© 2023 Gaffney A et al. *JAMA Network Open*.

To model the impact of altered management of PPROM on maternal morbidity, we first estimated the number of PPROM events in 2019 by multiplying a published prevalence estimate (0.37%)[42] by the number of births in 2019 for each state per CDC WONDER.[34] We then estimated PPROM morbidity in each state pre-ban by multiplying the number of PPROM events per state by the proportion of pregnant individuals with this condition treated expectantly (51.9%) vs. with termination (48.1%) and then by the corresponding morbidity rate with each approach (60.2%) and (33.0%), according to Sklar et al.'s estimates.[41] We then assumed that post-ban, 100% of previable PPROM management would be treated expectantly in abortion ban states, and 0% treated with initial termination, and re-calculated the morbidity rate under this scenario. Finally, we subtracted post-ban from pre-ban figures to estimate the increase in pregnancy-related morbidity stemming from altered management of PPROM. We took the same approach to calculate the increase in postpartum hemorrhage for such patients annually.

© 2023 Gaffney A et al. *JAMA Network Open*.

eFigure 1. Deaths Among Nonvaccinated Adults Aged 18 to 64 Years With and Without Implementation of the Occupational Safety and Health Administration Emergency Temporary Standard (ETS) in 3 Scenarios

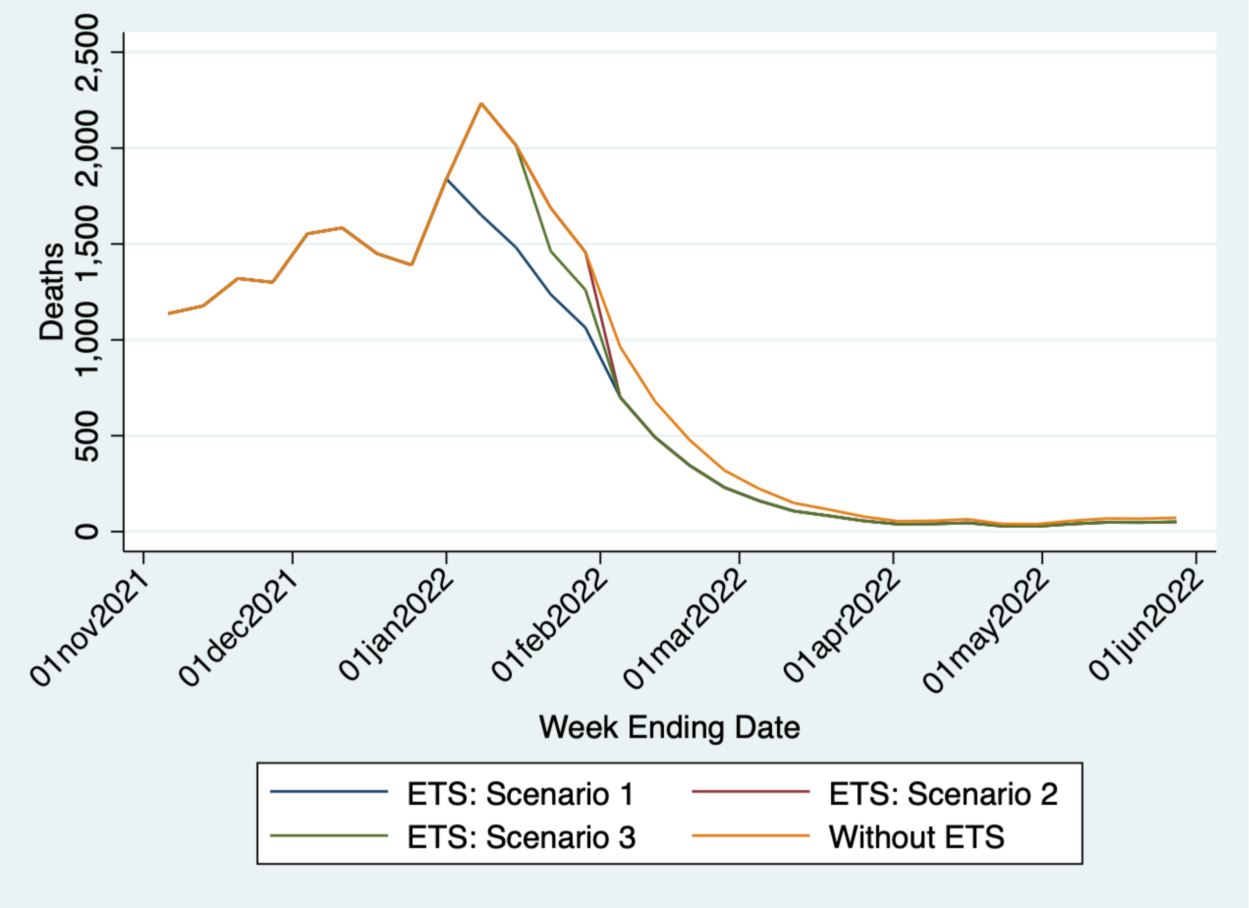

© 2023 Gaffney A et al. *JAMA Network Open*.

eTable 1. Policy Responses of May-Issue [Firearm Carry License] States Following the *Bruen* Decision

"May Issue" States	Policy Responses
California	Guidance from the state attorney general noted that firearm licensing authorities and law enforcement could not continue to require "good cause" prior to issuance of a carry permit, although requirements to show "good moral character" could continue to be enforced.[43] New laws passed in the wake of the decision increased potential legal culpability of firearms manufacturers, further restricted firearms advertising to young people, and added more restrictions to "ghost guns," although these are expected to undergo court challenge.[44]
Hawaii	The attorney general instructed law enforcement to grant concealed carry licenses without requiring that applicants are an "exceptional case."[45]
Maryland	The governor responded to the decision by removing restrictions on access to concealed carry permits.[46] According to his statement:<br><br>*"Last month, the U.S. Supreme Court struck down a provision in New York law pertaining to handgun permitting that is virtually indistinguishable from Maryland law. In light of the ruling and to ensure compliance with the Constitution, I am directing the Maryland State Police to immediately suspend utilization of the 'good and substantial reason' standard when reviewing applications for Wear and Carry Permits. It would be unconstitutional to continue enforcing this provision in state law. There is no impact on other permitting requirements and protocols."*[47]<br><br>However, Democratic lawmakers are pushing forward a bill to expand gun regulation.
Massachusetts	The Massachusetts Attorney General issued guidance instructing licensing authorities to stop enforcing the "good reason" requirement of the state's license-to-carry law, but instructing them to continue enforcement of "suitability" requirements. In response to guidance from the state, police are removing constraints on carry for a restricted licenses in some communities[48] Meanwhile, Democratic lawmakers are trying to pass legislation that would expand firearm regulation while, they say, ensuring compliance with *Bruen*.[49]
New York	Passed an aggressive gun control law creating numerous relatively onerous requirements for a carry permit, including provision of social media accounts, 16-hours of in-person training, re-certification every 3 years, expansion of "sensitive places" where guns are excluded, banning concealed carry in private residents without explicit permission from the owner, and several other restrictions.[50] However, it was widely assumed that these provisions would face court challenge,[50] and indeed in early October a federal court declared some but not all of the provisions unconstitutional.[20]

© 2023 Gaffney A et al. *JAMA Network Open*.

New Jersey	In the wake of the ruling and the mass shooting in Uvalde, TX, the governor of New Jersey signed 7 bills into law, including laws to: require firearm training to purchase guns; ban future sale of 0.50 caliber rifles; eventually require sale of micro-stamped firearms; require reporting of ammunition; sales heighten potential penalties for production of ghost guns; and several other measures.[51] Meanwhile, other state gun laws were facing legal challenges.[52]

Sources include those referenced in the table as well as reporting from *The Trace*.[53]

© 2023 Gaffney A et al. *JAMA Network Open*.

eTable 2. Studies Examining Impacts of Shall-Issue [License to Carry] (or Related) Right-to-Carry State Provisions

Publication	Summary	Interpretation
Donahue et al., 2022[21]	In this recent analysis, Donahue and colleagues again use panel data from 1979 to 2019 to examine the mechanisms by which "right to carry" (RTC) laws lead to more violent crime, looking at gun loss/theft and police effectiveness. They use data from the FBI Uniform Crime Reporting and Supplementary Homicide Reports, and perform a city-level panel data analysis. They first look at impacts on crime, and find that crime rises by 11-15% with introduction of RTC, with significant increase in firearm-related violent crime, firearm-related robbery, and firearm-related aggravated assault. There is a 13% increase in firearm-related homicide offset by a 3.43% fall in nonfirearm-related homicide (an overall 8.5% increase), but none of those estimates are significant. They then proceed to look at potential mechanisms for such effects. They find that crime clearance rates fall by 7.5-15% as a result of RTC laws, which is not explainable by the increase in crime itself. Next, they find that RTC laws are associated with a 35% increase in the dollar value of stolen guns per capita. This suggests that the number of stolen guns would increase by about 100,000 (in 2015) as a consequence of RTC laws, which is similar to a survey based estimate from their earlier work. They argue that previous studies failed to account for these sorts of unintended consequences of RTC laws.	Provides robust evidence, similar to Donohue 2019, of association between RTC laws and violent crime. There is a 8.5% overall increase in homicide, but the effect estimate is not statistically significant.
Doucette et al.[54]	This analysis employed a quasi-experimental design examining the association between adoption of "shall-issue" laws and state-level violent crime, with national data from 1980-2019. Demonstrated that the implementation of shall-issue laws was associated with a 9.5% increase in gun assaults, as well as an 8.8% increase in non-gun homicides; the increase in gun homicides did not reach statistical significance. Effects appeared larger in states without violent misdemeanor prohibition provisions.	Provides further evidence of effect of "shall issue" laws and firearm assaults, finding a 10% increase.
Schell et al., 2020[22]	Schell et al. begin by noting that various studies have yielded varying results and conclusions, even when using the same data, suggesting that some methods may not have been appropriate. Proceeded with a simulation to examine how different methodologies compare in terms	Provides evidence for impacts of RTC on firearm-related deaths, with a 3% estimated effect relative to a more restrictive baseline.

	of modelling the impact of state laws on gun access/use and outcomes, and then used their "more appropriate statistical approach" to look at association of child access prevention (CAP), RTC, and stand your ground (SYG) laws on firearm deaths. Data was from 50 states for 1980-2016, with state-level firearm death data from the Vital Statistics System (VSS). The looked at 6 years after implementation, and used a Bayesian methodology. They find that CAP are associated with a 6% decline in firearm deaths, RTC with a 3 % increase, and SYG with a 3% increase. Together, taking a more restrictive approach on these 3 gun regulation issues could reduce firearm deaths by 11%.	
Donahue et al, 2019[55]	This analysis used two different methodologies to examine the impact of RTC laws in the US.<br><br>First, using panel data, the analysts conducted a simple difference-in-differences analyses, and found that from 1977 to 2014 there was a 42.3% drop in violent crime in states that never adopted RTC laws, versus a 4.3% drop among states that adopted such laws between 1977 and 2014 and a 9.9% drop among states that had adopted them before 1977. They then performed a panel data analysis for the full 1977-2014 period using two different models. Their own preferred models includes various demographic variables as well as variables for police and incarceration. This model finds a significant effect of RTC laws on increasing the violence crime rate (9.0%) and property crime rate (6.5%). There are non-significant and very imprecise effects for murder rate (2.27%) and firearm murder rate (2.90%). Note that one serious limitation of this study design is that it fails to account for spillover from RTC states to non-RTC states (i.e. in gun trafficking). They then proceed to repeat the analysis using the 36 co-variates employed in the study of Lott and Mustard (LM), which they argue is flawed and introduces statistical noise. They also show that the LM specification, but not their own, respects the parallel trends assumption. Moreover, when examining states that implemented RTC laws in the period 2000-2014 (following the end of the crack-cocaine epidemic), they found that both their model and the LM model show an increase in murder (and firearm murder) due to RTC laws *(Appendix C)*.	Provides robust evidence for RTC restrictions on violent crime. Impacts on homicide are less precise, and mostly not significant overall, although do emerge more clearly in the post-2000 panel data analysis.<br><br>Strengths: Robust causal inference methodology; recent data through 2014.

© 2023 Gaffney A et al. *JAMA Network Open*.

	Next, they examine the impact of RTC laws using a "synthetic controls" approach. Using this approach, they find that 31 states which adopted RTC laws experienced a mean effect after 10 years of a 14.3 percent increase in violent crime. The effect on murder is less precise and not significantly significant in the synthetic controls model. They find that the synthetic controls approach shows an estimated increase in murder of 8.7% (p<0.1) and in firearm murder of 15.3%.	
Siegel et al., 2019[23]	This analysis used the State Firearm Law Database, compiled by the authors, which provides state-year level data on state firearm laws. It used CDC Wonder for gun-related mortality at the state and annual level (1991-2016). It examined 10 state laws, including "shall issue" laws, and lagged the effects by one year. They used a DiD approach, as well as both state and year fixed effects, while controlling for 12 other state-level factors. When examined individually, universal background checks, violent misdemeanor laws, and "shall issue" laws were associated with higher homicide. However, controlling for all 10 laws at the same time demonstrated an association between universal background checks and 14.9% lower overall homicide; an 18.1% lower homicide with violent misdemeanor law; and 9.0% higher homicide rates with "shall issue" rules. Seven other types of laws were not independently associated with homicide. In a falsification test, each of these laws were associated with firearm homicide but not non-firearm homicide. For suicide, four of 10 laws were separately associated with suicide but only bans on junk guns and permit-less carry were associated with suicide, and both laws failed the falsification test.	Provides evidence for adverse effect of "shall issue" laws on homicide but not suicide; point estimate: 9.0% higher homicide.<br><br>Strengths include: DiD approach; recent data (1991-2016); falsification test (non-firearm suicide).
Crifasi et al., 2018[24]	The authors performed interrupted time series analysis examining effect of gun laws on homicide, from 1984-2015 in urban counties. They used CDC WONDER data for gun homicide, and included several state-level controls. They researched state laws and created month/day/year specific indicator variables for implementation of policies and found an association between permit-to-purchase (PTP) laws and a 14% reduction in firearm homicides; comprehensive background check-only laws and a 16% increase in firearm homicides; RTC and a 4% increase in firearm homicides; SYG and a 7% increase in firearm	Provides evidence for beneficial effect of "permit-to-purchase" laws on homicide. Did not examine suicide.<br><br>Point estimate: 14% lower homicide with permit-to-purchase homicide. 4% higher with right to carry laws.

© 2023 Gaffney A et al. *JAMA Network Open*.

	homicides; violent misdemeanor prohibitions and a 14% increase in firearm homicides. None of the laws had an impact on non-firearm homicide. They suggest that their estimates for the effect of laws excluding those with violent misdemeanors from gun purchases may be biased.	Limitations: Inconsistent results (with harm apparent with some laws) suggesting potential bias in design.
Siegel et al., 2017[56]	These researchers used CDC WISQARS database for age-adjusted homicide rates overall (1991-2015) and FBI Uniform Crime Reports data for handgun vs. long-gun homicide. They constructed a state law database to look at effect of "shall issue" provisions, controlling for 12 state-level factors associated homicide, as well as other state firearm laws apart from "may issue" vs. "shall issue." They found that "shall issue" concealed-carry laws were associated with 6.5% higher homicide vs. "may issue" states. This effect held for firearm homicide rates (but not non-firearm suicide), and for handgun homicide (but not long-gun homicide) and was robust to various other specifications.	Provides evidence for adverse effect of "shall issue" laws on homicide. Did not examine suicide.<br><br>Point estimate: 6.5% higher homicide, 8.6% higher firearm homicide, and 10.6% higher handgun homicide.<br><br>Strengths include: well controlled; recent data; two falsification tests (non-firearm homicide and long-gun homicide).

© 2023 Gaffney A et al. *JAMA Network Open*.

eTable 3. Effect of *Bruen* on State Firearm Death Rates Under Differing State Policy Responses and Assumptions About Right-to-Carry Law Effects

		"Expected outcome" from RTC Legislation		
		Low (3% Increase)	Mid (6% Increase)	High (9% Increase)
State Policy Response	Fully Offset (0%)	0%	0%	0%
	Partially Offset (50%)	1.5%	3%	4.5%
	No Offset (100%)	3%	6%	9%

Note: Each cell represents a potential relative increase in firearm-related mortality due to *Bruen*, and is the product of the assumed state policy response (0-100%, seen in the row labels) and the "expected outcome" from RTC legislation estimates (3-9%, seen in the column headers).

© 2023 Gaffney A et al. *JAMA Network Open*.

eTable 4. Estimated Effects of Implementation of the Occupational Safety and Health Administration Emergency Temporary Standard (ETS) Midpoint Estimate (Primary Scenario)

A	B	C	D	E	F	G	H
Week Ending Date	Unvacci-nated population, 18-64	Number vaccinated due to ETS	Share vaccinated due to ETS *(= C/B)*	Estimated Deaths Among Non-Elderly Adults Unvaccinated	Deaths averted, unadjusted *(=D*E)*	Deaths averted, final *(=F* 0.85)*	Unvaccinated deaths with ETS *(=E-G)*
1/1/22	62,223,648	-	0.00	1,839	-	-	1,839
1/8/22	61,569,584	-	0.00	2,234	-	-	2,234
1/15/22	60,794,816	-	0.00	2,015	-	-	2,015
1/22/22	60,163,392	9,457,264	0.16	1,688	265	226	1,462
1/29/22	59,562,192	9,457,264	0.16	1,457	231	197	1,260
2/5/22	59,020,288	18,914,528	0.32	963	309	262	701
2/12/22	58,492,976	18,914,528	0.32	679	220	187	492
2/19/22	57,941,904	18,914,528	0.33	477	156	132	345
2/26/22	57,579,632	18,914,528	0.33	319	105	89	230
3/5/22	57,241,248	18,914,528	0.33	223	74	63	160
3/12/22	56,970,000	18,914,528	0.33	149	49	42	107
3/19/22	56,736,256	18,914,528	0.33	115	38	33	82
3/26/22	56,542,768	18,914,528	0.33	78	26	22	56
4/2/22	56,334,528	18,914,528	0.34	54	18	15	39
4/9/22	56,067,648	18,914,528	0.34	56	19	16	40
4/16/22	55,810,160	18,914,528	0.34	64	22	18	46
4/23/22	55,606,368	18,914,528	0.34	40	14	12	28

© 2023 Gaffney A et al. *JAMA Network Open*.

A	B	C	D	E	F	G	H
Week Ending Date	Unvacci-nated population, 18-64	Number vaccinated due to ETS	Share vaccinated due to ETS	Estimated Deaths Among Non-Elderly Adults Unvaccinated	Deaths averted, unadjusted	Deaths averted, final	Unvaccinated deaths with ETS
4/30/22	55,415,584	18,914,528	0.34	39	13	11	28
5/7/22	55,206,336	18,914,528	0.34	56	19	16	40
5/14/22	54,937,296	18,914,528	0.34	68	23	20	48
5/21/22	54,793,984	18,914,528	0.35	67	23	20	47
5/28/22	54,610,256	18,914,528	0.35	72	25	21	51
			Total Deaths:	***12,752***	***1,649***	***1,402***	***11,350***

© 2023 Gaffney A et al. *JAMA Network Open*.

eTable 5. Annual Increase in the Number of Firearm-Related Homicides in 6 May-Issue States as a Result of *Bruen*, All Scenarios

State	2020 Firearm Mortality	Assumed relative increase in firearm-related mortality					
		0%*	1.5%	3%	4.5%**	6%	9%***
California	1,732	0	26	52	78	104	156
Hawaii	16	0	0	0	1	1	1
Maryland	526	0	8	16	24	32	47
Massachusetts	130	0	2	4	6	8	12
New York	561	0	8	17	25	34	50
New Jersey	253	0	4	8	11	15	23
District of Columbia	157	0	2	5	7	9	14
**Total**	*3,375*	*0*	*51*	*101*	*152*	*203*	*304*

Note: 2020 Firearm Homicide is from CDC WONDER, Underlying Cause of Death file.
* = lower bound estimate
** = mid/primary estimate
*** = upper bound estimate

© 2023 Gaffney A et al. *JAMA Network Open*.

eTable 6. Annual Increase in the Number of Firearm-Related Nonfatal Injuries Resulting in Significant Body Region Injury*

Body Region	Percent with significant regional injury among those with nonfatal injuries[26]	Scenarios*		
		Low Bound	Primary Scenario	High Bound
Head/Neck	7.7%	0	29	58
Chest	13.5%	0	51	102
Abdomen/pelvis	9.7%	0	37	73
Extremity	20.4%	0	77	154

* We defined "significant" body region damage as body region injury of abbreviated injury scale >=2.
** Lower bound = 0% change in firearm-related mortality; primary scenario = 4.5% increase in firearm mortality; upper bound = 9% increase in firearm mortality.

© 2023 Gaffney A et al. *JAMA Network Open*.

eTable 7. Annual Increase in Morbidity Due to Changed Management of Previable Premature Rupture of Membranes as a Result of *Dobbs v Jackson Women's Health Organization*

	Abortion Bans States with Current Bans only	Abortion Bans in States with Current Bans + in "High Risk" States
Composite Morbidity	454	826
Postpartum Hemorrhage	202	367

© 2023 Gaffney A et al. *JAMA Network Open*.

eReferences

1. Department of Labor; Occupational Safety and Health Administraiton. COVID-19 Vaccination and Testing; Emergency Temporary Standard. *Federal Register*. 2021;86(112). https://www.govinfo.gov/content/pkg/FR-2021-11-05/pdf/2021-23643.pdf

2. Hirsch L. U.S. Sets Jan. 4 Vaccination Deadline for Big Private Employers. *The New York Times*. https://www.nytimes.com/2021/11/04/business/biden-vaccine-mandate-osha.html. Published November 4, 2021. Accessed August 2, 2022.

3. Savage C. U.S. Urges Court Not to Block Vaccine Mandate on Employers. *The New York Times*. https://www.nytimes.com/2021/11/08/us/politics/employer-vaccine-mandates.html. Published November 9, 2021. Accessed August 2, 2022.

4. Savage C. Appeals Court Extends Block on Biden's Vaccine Mandate for Employers. *The New York Times*. https://www.nytimes.com/2021/11/12/us/politics/court-vaccine-mandate.html. Published November 13, 2021. Accessed August 2, 2022.

5. Savage C. Challenges to Workplace Vaccine Mandate Moved to Appeals Court in Cincinnati. *The New York Times*. https://www.nytimes.com/2021/11/16/us/politics/biden-vaccine-mandate-osha-ohio.html. Published November 16, 2021. Accessed August 2, 2022.

6. Hirsch L, Goldberg E, Savage C. Appeals Court Reinstates OSHA's Vaccine Mandate for Workers at Larger Businesses. *The New York Times*. https://www.nytimes.com/2021/12/17/business/osha-vaccine-mandate.html. Published December 18, 2021. Accessed August 2, 2022.

7. OSHA. Health Impacts of the COVID-19 Vaccination and Testing ETS.

8. Occupational Safety and Health Administration (OSHA). Analytical Spreadsheets in Support of the COVID-19 Vaccination and Testing ETS. Published October 2021a. Accessed August 2, 2022. https://www.regulations.gov/document/OSHA-2021-0007-0486

9. Centers for Disease Control and Prevention. Rates of COVID-19 Cases or Deaths by Age Group and Vaccination Status. Accessed August 8, 2022. https://data.cdc.gov/Public-Health-Surveillance/Rates-of-COVID-19-Cases-or-Deaths-by-Age-Group-and/3rge-nu2a

10. AH Provisional COVID-19 Death Counts by Week, Race, and Age, United States 2020-2022 | Data | Centers for Disease Control and Prevention. Accessed August 9, 2022. https://data.cdc.gov/NCHS/AH-Provisional-COVID-19-Death-Counts-by-Week-Race-/siwp-yg6m

11. Centers for Disease Control and Prevention. COVID-19 Vaccinations in the United States, Jurisdiction. Accessed August 3, 2022. https://data.cdc.gov/Vaccinations/COVID-19-Vaccinations-in-the-United-States-Jurisdi/unsk-b7fc

© 2023 Gaffney A et al. *JAMA Network Open*.

12. Bureau UC. National Population by Characteristics: 2020-2021. Census.gov. Accessed August 9, 2022. https://www.census.gov/data/tables/time-series/demo/popest/2020s-national-detail.html

13. Tenforde MW. Effectiveness of mRNA Vaccination in Preventing COVID-19–Associated Invasive Mechanical Ventilation and Death — United States, March 2021–January 2022. *MMWR Morb Mortal Wkly Rep*. 2022;71. doi:10.15585/mmwr.mm7112e1

14. CDC. Cases, Data, and Surveillance. Centers for Disease Control and Prevention. Published February 11, 2020. Accessed August 10, 2022. https://www.cdc.gov/coronavirus/2019-ncov/covid-data/covid-net/purpose-methods.html

15. The Supreme Court knocked back blue states on gun restrictions. They're seeing how far they can step forward. POLITICO. Accessed August 2, 2022. https://www.politico.com/news/2022/07/08/blue-states-test-limits-of-gun-laws-after-supreme-court-raises-the-bar-00044486

16. The Real Significance of the Supreme Court's Gun Decision. The Trace. Published July 19, 2022. Accessed August 2, 2022. https://www.thetrace.org/2022/07/the-real-significance-of-the-supreme-courts-gun-decision/

17. Ulrich MR. Public Carry versus Public Health — The Harms to Come from the Supreme Court's Decision in Bruen. *N Engl J Med*. 2022;387(14):1245-1247. doi:10.1056/NEJMp2210269

18. Charles J. Bruen, Analogies, and the Quest for Goldilocks History. Duke Center for Firearms Law. Published June 28, 2022. Accessed August 1, 2022. https://firearmslaw.duke.edu/2022/06/bruen-analogies-and-the-quest-for-goldilocks-history/

19. School SL. Packed and Loaded: Stanford's John Donohue on Supreme Court's Guns Decision. Stanford Law School. Accessed August 1, 2022. https://law.stanford.edu/2022/06/24/packed-and-loaded-stanfords-john-donohue-on-supreme-courts-guns-decision/

20. Bromwich JE. Federal Judge Blocks N.Y. Gun Law, Finding Much of It Unconstitutional. *The New York Times*. https://www.nytimes.com/2022/10/06/nyregion/judge-blocks-ny-gun-law.html. Published October 6, 2022. Accessed October 8, 2022.

21. Donohue JJ, Cai SV, Bondy MV, Cook PJ. *More Guns, More Unintended Consequences: The Effects of Right-to-Carry on Criminal Behavior and Policing in US Cities*. National Bureau of Economic Research; 2022. doi:10.3386/w30190

22. Schell TL, Cefalu M, Griffin BA, Smart R, Morral AR. Changes in firearm mortality following the implementation of state laws regulating firearm access and use. *Proceedings of the National Academy of Sciences*. 2020;117(26):14906-14910. doi:10.1073/pnas.1921965117

© 2023 Gaffney A et al. *JAMA Network Open*.

23. Siegel M, Pahn M, Xuan Z, Fleegler E, Hemenway D. The Impact of State Firearm Laws on Homicide and Suicide Deaths in the USA, 1991–2016: a Panel Study. *J Gen Intern Med*. 2019;34(10):2021-2028. doi:10.1007/s11606-019-04922-x

24. Crifasi CK, Merrill-Francis M, McCourt A, Vernick JS, Wintemute GJ, Webster DW. Association between Firearm Laws and Homicide in Urban Counties. *J Urban Health*. 2018;95(3):383-390. doi:10.1007/s11524-018-0273-3

25. Underlying Cause of Death, 1999-2020 Request Form. Accessed September 27, 2022. https://wonder.cdc.gov/controller/datarequest/D76

26. Kaufman EJ, Wiebe DJ, Xiong RA, Morrison CN, Seamon MJ, Delgado MK. Epidemiologic Trends in Fatal and Nonfatal Firearm Injuries in the US, 2009-2017. *JAMA Internal Medicine*. 2021;181(2):237-244. doi:10.1001/jamainternmed.2020.6696

27. Myers C, Jones R, Upadhyay U. Predicted changes in abortion access and incidence in a post-Roe world. *Contraception*. 2019;100(5):367-373. doi:10.1016/j.contraception.2019.07.139

28. Lindo JM, Myers C, Schlosser A, Cunningham S. How Far Is Too Far? New Evidence on Abortion Clinic Closures, Access, and Abortions. Published online April 2017. doi:10.3386/w23366

29. Myers C. Myers Abortion Facility Database. Published online July 16, 2021. doi:10.17605/OSF.IO/8DG7R

30. Myers CK. Measuring the Burden: The Effect of Travel Distance on Abortions and Births. *SSRN Journal*. Published online 2021. doi:10.2139/ssrn.3892584

31. Nash E, Guarnier I. Six Months Post-Roe, 24 US States Have Banned Abortion or Are Likely to Do So: A Roundup. Guttmacher Institute. Published January 9, 2023. Accessed April 7, 2023. https://www.guttmacher.org/2023/01/six-months-post-roe-24-us-states-have-banned-abortion-or-are-likely-do-so-roundup

32. Stevenson AJ. The Pregnancy-Related Mortality Impact of a Total Abortion Ban in the United States: A Research Note on Increased Deaths Due to Remaining Pregnant. *Demography*. 2021;58(6):2019-2028. doi:10.1215/00703370-9585908

33. National Center for Health Statistics. Maternal deaths and mortality rates: Each state, the District of Columbia, United States, 2018-2020. https://www.cdc.gov/nchs/maternal-mortality/MMR-2018-2020-State-Data.pdf

34. CDC WONDER. Accessed September 21, 2022. https://wonder.cdc.gov/

35. Guttmacher Data Center. Abortions by state of residence: Number of abortions. Accessed October 8, 2022. https://data.guttmacher.org/states/table?state=AL+AK+AZ+AR+CA+CO+CT+DE+DC+FL+GA+HI+ID+IL+IN+IA+KS+KY+LA+ME+MD+MA+MI+MN+MS+MO+MT+NE+NV+

© 2023 Gaffney A et al. *JAMA Network Open*.

NH+NJ+NM+NY+NC+ND+OH+OK+OR+PA+RI+SC+SD+TN+TX+UT+VT+VA+WA+WV+WI+WY&topics=69&dataset=data

36. Reale SC, Easter SR, Xu X, Bateman BT, Farber MK. Trends in Postpartum Hemorrhage in the United States From 2010 to 2014. *Anesthesia & Analgesia*. 2020;130(5):e119. doi:10.1213/ANE.0000000000004424

37. Arey W, Lerma K, Beasley A, Harper L, Moayedi G, White K. A Preview of the Dangerous Future of Abortion Bans — Texas Senate Bill 8. *N Engl J Med*. 2022;387(5):388-390. doi:10.1056/NEJMp2207423

38. Méndez RO and M. Texas hospitals are putting pregnant patients at risk by denying care out of fear of abortion laws, medical group says. The Texas Tribune. Published July 15, 2022. Accessed August 8, 2022. https://www.texastribune.org/2022/07/15/texas-hospitals-abortion-laws/

39. Some Abortion Bans Put Patients, Doctors at Risk in Emergencies. Accessed September 21, 2022. https://pew.org/3eea3RG

40. Nambiar A, Patel S, Santiago-Munoz P, Spong CY, Nelson DB. Maternal morbidity and fetal outcomes among pregnant women at 22 weeks' gestation or less with complications in two Texas hospitals after legislation on abortion. *American Journal of Obstetrics and Gynecology*. Published online July 2022:S0002937822005361. doi:10.1016/j.ajog.2022.06.060

41. Sklar A, Sheeder J, Davis AR, Wilson C, Teal SB. Maternal morbidity after preterm premature rupture of membranes at <24 weeks' gestation. *Am J Obstet Gynecol*. 2022;226(4):558.e1-558.e11. doi:10.1016/j.ajog.2021.10.036

42. Waters TP, Mercer BM. The management of preterm premature rupture of the membranes near the limit of fetal viability. *Am J Obstet Gynecol*. 2009;201(3):230-240. doi:10.1016/j.ajog.2009.06.049

43. legal-alert-oag-2022-02.pdf. Accessed August 2, 2022. https://oag.ca.gov/system/files/media/legal-alert-oag-2022-02.pdf

44. Twitter, Instagram, Email, Facebook. California enacts sweeping gun control laws, setting up a legal showdown. Los Angeles Times. Published July 12, 2022. Accessed August 2, 2022. https://www.latimes.com/california/story/2022-07-12/gavin-newsom-signs-firearm-laws-supreme-court-gun-rights

45. Hawaii AG Instructs Chiefs of Police to Broadly Grant Concealed Carry Licenses, but not Open Carry. Reason.com. Accessed August 2, 2022. https://reason.com/volokh/2022/07/11/hawaii-ag-instructs-chiefs-of-police-to-broadly-grant-concealed-carry-licenses-but-not-unconcealed-carry/

46. Maryland moves to loosen concealed carry gun law after Supreme Court ruling. Restoring America. Published July 6, 2022. Accessed August 2, 2022.

© 2023 Gaffney A et al. *JAMA Network Open*.

https://www.washingtonexaminer.com/restoring-america/faith-freedom-self-reliance/maryland-moves-to-loosen-concealed-carry-gun-law-after-supreme-court-ruling

47. Governor Larry Hogan - Official Website for the Governor of Maryland. Governor of Maryland. Accessed August 1, 2022. http://Governor.maryland.gov

48. Staff MSG, July 17 U, 2022, Comments169 5:46 p m Share on Facebook Share on TwitterView. Boston, Springfield begin to lift gun license restrictions after Supreme Court decision - The Boston Globe. BostonGlobe.com. Accessed August 1, 2022. https://www.bostonglobe.com/2022/07/17/metro/boston-springfield-begin-lift-gun-license-restrictions-after-supreme-court-decision/

49. Staff MSG, July 21 U, 2022, Comments96 7:24 p m Share on Facebook Share on TwitterView. Mass. House swiftly passes changes to firearms law, aiming to protect state's strict gun control regime after Supreme Court decision - The Boston Globe. BostonGlobe.com. Accessed August 1, 2022. https://www.bostonglobe.com/2022/07/21/metro/after-supreme-court-ruling-mass-lawmakers-scramble-tighten-states-gun-laws/

50. Willinger A. New York's Response to Bruen: The Outer Limits of the "Sensitive Places" Doctrine | Duke Center for Firearms Law. Published July 13, 2022. Accessed August 1, 2022. https://firearmslaw.duke.edu/2022/07/new-yorks-response-to-bruen-the-outer-limits-of-the-sensitive-places-doctrine/

51. NJ.com BJ| NAM for. Murphy signs 7 new laws to tighten N.J. gun regulations, vows more action on firearms. nj. Published July 5, 2022. Accessed August 2, 2022. https://www.nj.com/politics/2022/07/murphy-signs-7-new-laws-to-tighten-nj-gun-regulations-vows-more-action-on-firearms.html

52. NJ.com SPS| NAM for. N.J. gun laws face new legal challenges after Supreme Court strikes down concealed carry law. nj. Published July 7, 2022. Accessed August 2, 2022. https://www.nj.com/politics/2022/07/nj-gun-laws-face-new-legal-challenges-after-supreme-court-strikes-down-concealed-carry-law.html

53. Tracking the Effects of the Supreme Court's Gun Ruling. The Trace. Published August 1, 2022. Accessed August 2, 2022. https://www.thetrace.org/2022/08/nysrpa-v-bruen-challenge-gun-regulations/

54. Doucette ML, McCourt AD, Crifasi CK, Webster DW. Impact of Changes to Concealed Carry Weapons Laws on Fatal and Nonfatal Violent Crime, 1980-2019. *Am J Epidemiol*. Published online September 14, 2022:kwac160. doi:10.1093/aje/kwac160

55. Donohue JJ, Aneja A, Weber KD. Right-to-Carry Laws and Violent Crime: A Comprehensive Assessment Using Panel Data and a State-Level Synthetic Control Analysis. *Journal of Empirical Legal Studies*. 2019;16(2):198-247. doi:10.1111/jels.12219

© 2023 Gaffney A et al. *JAMA Network Open*.

56. Siegel M, Xuan Z, Ross CS, et al. Easiness of Legal Access to Concealed Firearm Permits and Homicide Rates in the United States. *Am J Public Health*. 2017;107(12):1923-1929. doi:10.2105/AJPH.2017.304057

© 2023 Gaffney A et al. *JAMA Network Open*.
