# Supplementary material for: Projected Health Outcomes Associated With 3 US Supreme Court Decisions in 2022 on COVID-19 Workplace Protections, Handgun-Carry Restrictions, and Abortion Rights
Source: JAMA Netw Open. 2023 Jun 8;6(6):e2315578. doi: 10.1001/jamanetworkopen.2023.15578 (PMC10251209; doi:10.1001/jamanetworkopen.2023.15578)
Supplement: Supplement 2. — Data Sharing Statement [file jamanetwopen-e2315578-s002.pdf]

# Data Sharing Statement

Gaffney. Projected Health Outcomes Associated With 3 US Supreme Court Decisions in 2022 on COVID-19 Workplace Protections, Handgun-Carry Restrictions, and Abortion Rights. *JAMA Netw Open*. Published June 08, 2023. doi:10.1001/jamanetworkopen.2023.15578

**Data**
**Data available:** No
